# Supplementary material for: Engineering the thermal conductivity along an individual silicon nanowire by selective helium ion irradiation
Source: Nat Commun. 2017 Jun 27;8:15919. doi: 10.1038/ncomms15919 (PMC5490267; doi:10.1038/ncomms15919)
Supplement: Supplementary Information [file ncomms15919-s1.pdf]

Type of file: pdf
Size of file: 9,477 KB
Title of file for HTML: Supplementary Information
Description: Supplementary Figures, Supplementary Tables, Supplementary Notes and Supplementary References.

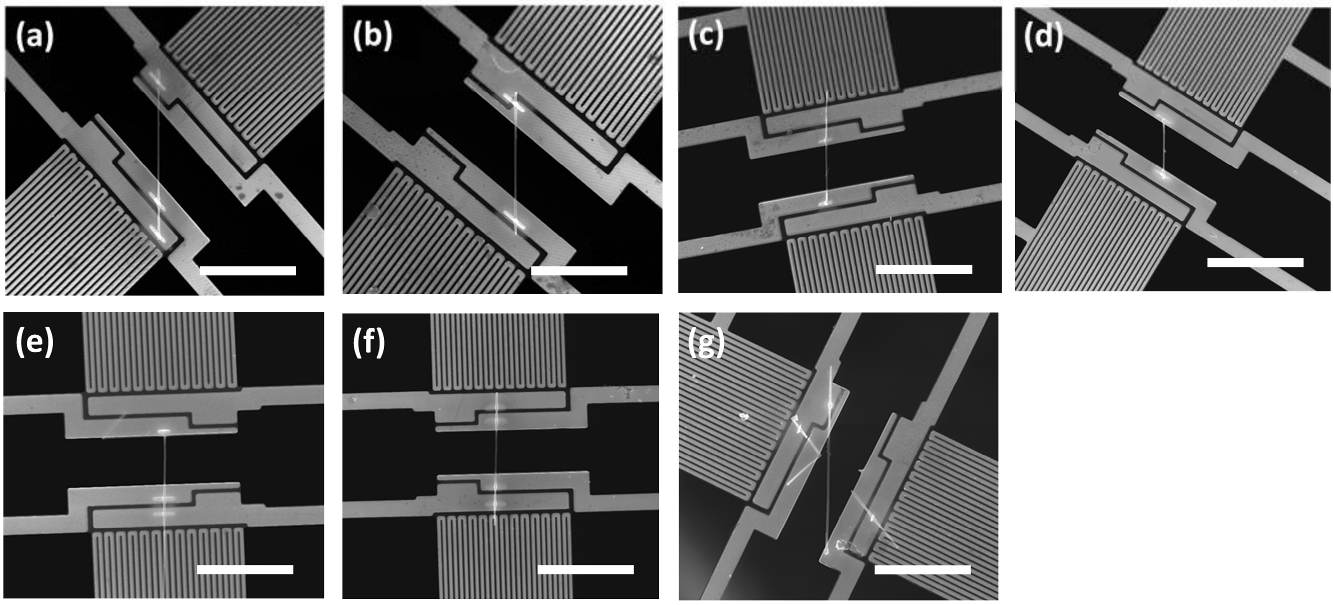

Supplementary Figure 1 | Silicon nanowires on METS device. (a)-(g) are SEM images of measured samples #2 to #8, respectively. The scale bars are 10 μm.

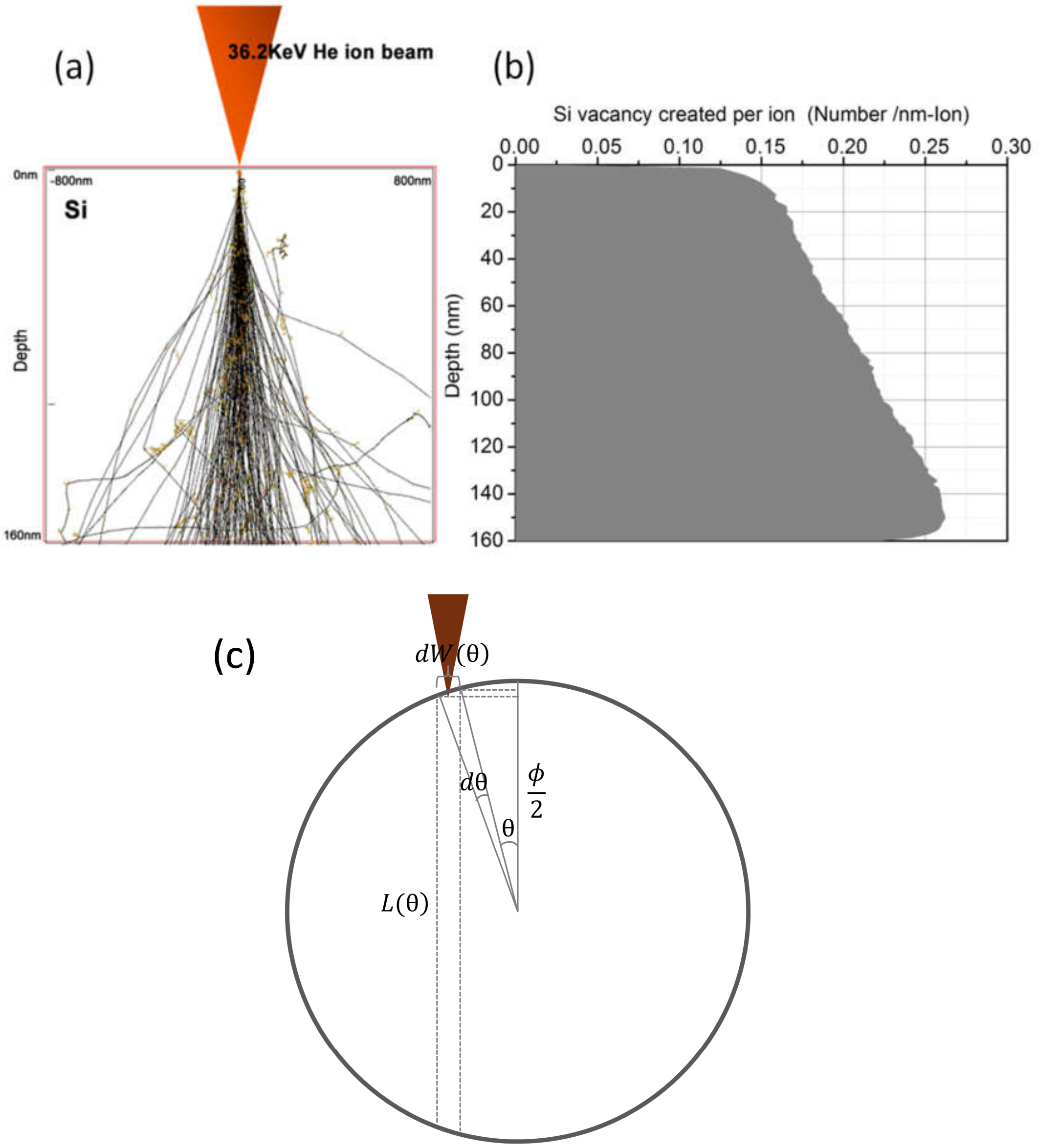

Supplementary Figure 2 | Monte Carlo calculation of lattice damage by a 36.2 keV He ion beam on a 160 nm thick Si film. (a) Solid black line: The trajectory of the helium ion. Red dots: Vacancies created by the helium ion and target atoms (Si in our case). Green dots: vacancies created by recoiling target atoms. (b) The damage creation in the Si film. Vacancy means that the target atoms are knocked off their lattice site and are *not* replaced by the other recoiling target atoms. (c) Sketch of helium ion travelling across the cross-section of silicon nanowire. ϕ is diameter of nanowire, $L(\theta)$ is the actual travelling trajectory length of helium ion and $\mathrm{d}W(\theta)$ is the horizontal element for integration at an angle of θ.

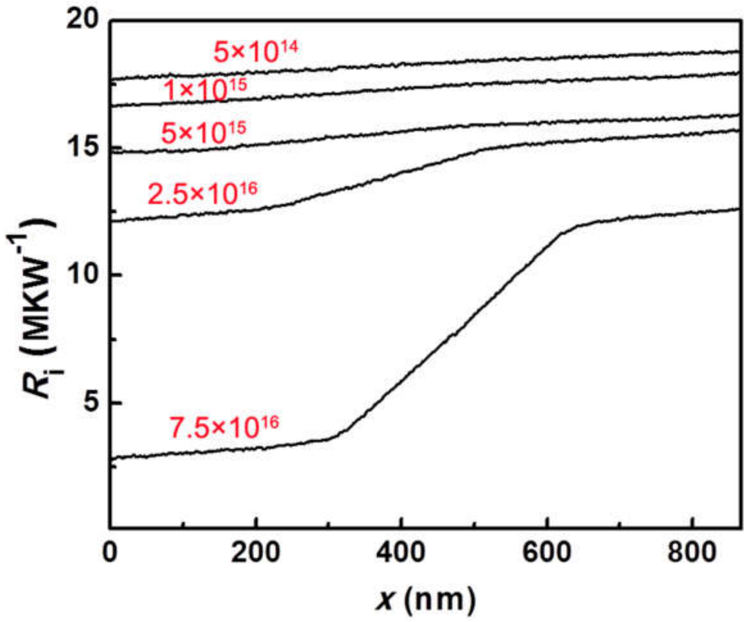

Supplementary Figure 3 | $R_i(x)$ curve for all the damaged portions for Sample #1. The dose in unit of cm^{-2} is labeled in red above each curve.

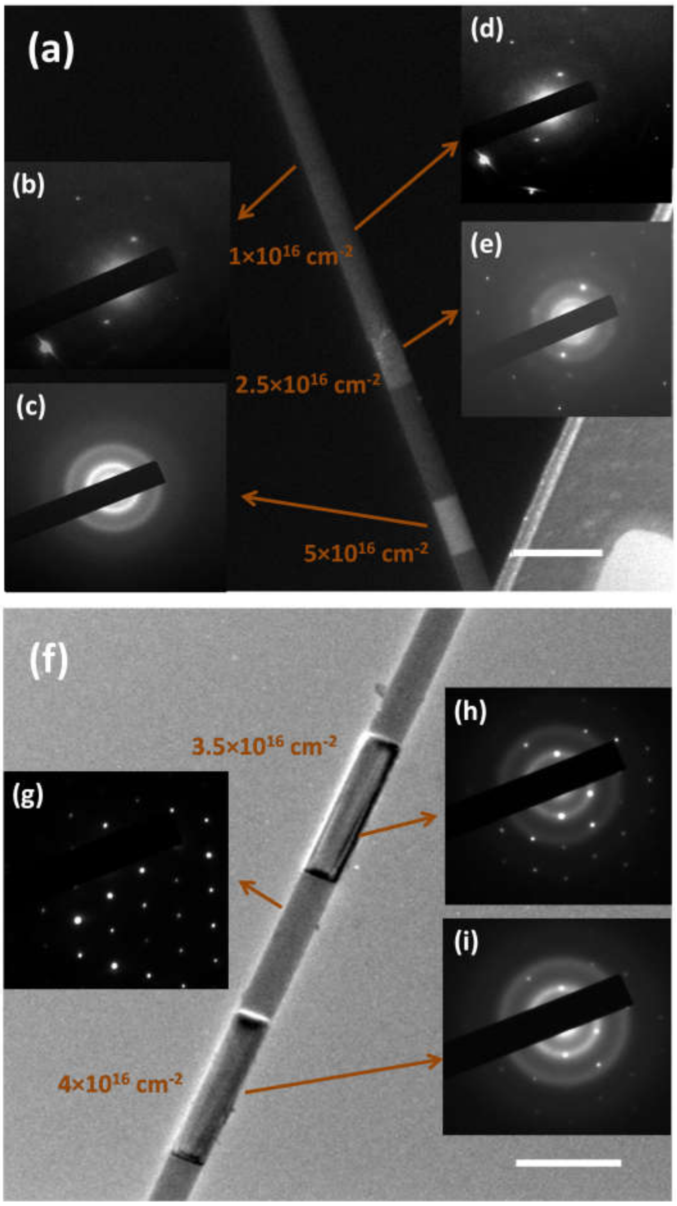

Supplementary Figure 4 |TEM image and the diffraction patterns. (a) TEM image of Sample #2. Inset (c), (d), (e) are the diffraction pattern of portions with helium dose of 5×10^{16}, 1×10^{16}, 2.5×10^{16} cm^{-2}, respectively. Inset (b) is the diffraction pattern of the non-irradiated portion, which is indistinguishable from that of (d), indicating that the crystallinity is still preserved. (f) TEM image and diffraction patterns of helium ion irradiated silicon nanowire. Inset (h) and (i) are diffraction pattern of portions with dose of 3.5×10^{16} and 4×10^{16} cm^{-2}, and (g) is for non-irradiated dose. With increasing dose, the Debye-Scherrer rings becomes stronger while the crystalline lattice becomes inconspicuous, until the final amorphization at even higher dose, which is corresponding to the decrease in the thermal conductivity. The scale bar for both (a) and (f) is 500nm.

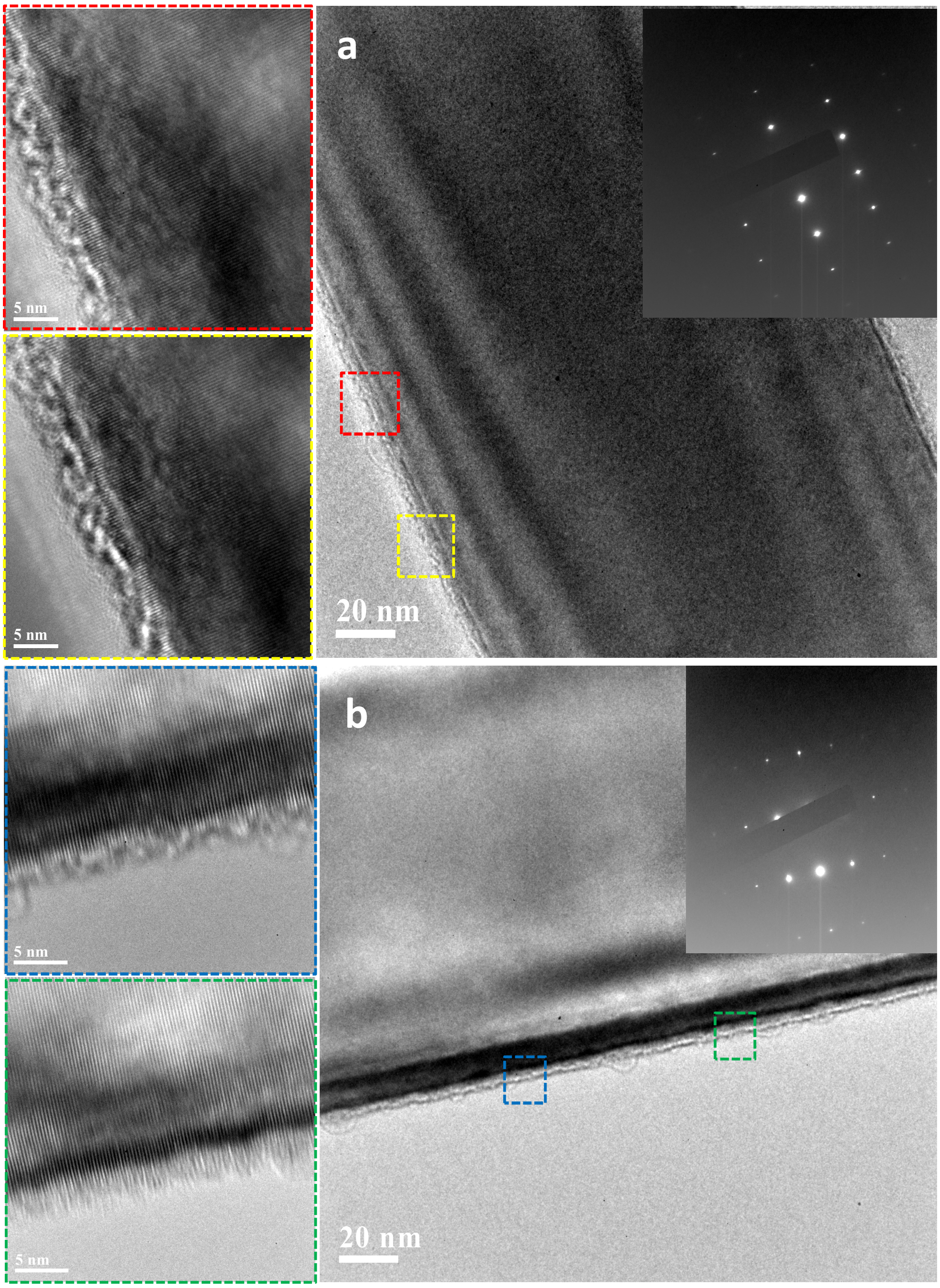

Supplementary Figure 5 | TEM characterization of two silicon nanowires after annealing (a) and (b). Inset is the selected area electron diffraction pattern. For (a) and (b), the left two figures are the high resolution TEM of selected areas that are marked by a certain color square along the silicon nanowire, from which, the nanowire is seen to remain single-crystalline rather than core/shell crystalline/amorphous-like structure after final annealing, which is corresponding to the thermal conductivity measurement of intrinsic silicon nanowire. The unavoidable intrinsic oxidation layer of the measured nanowires is ~2.5nm, which is similar to or even slightly smaller than that found in electroless-etching (EE) silicon nanowires[1]. Scale bar is shown in each figure.

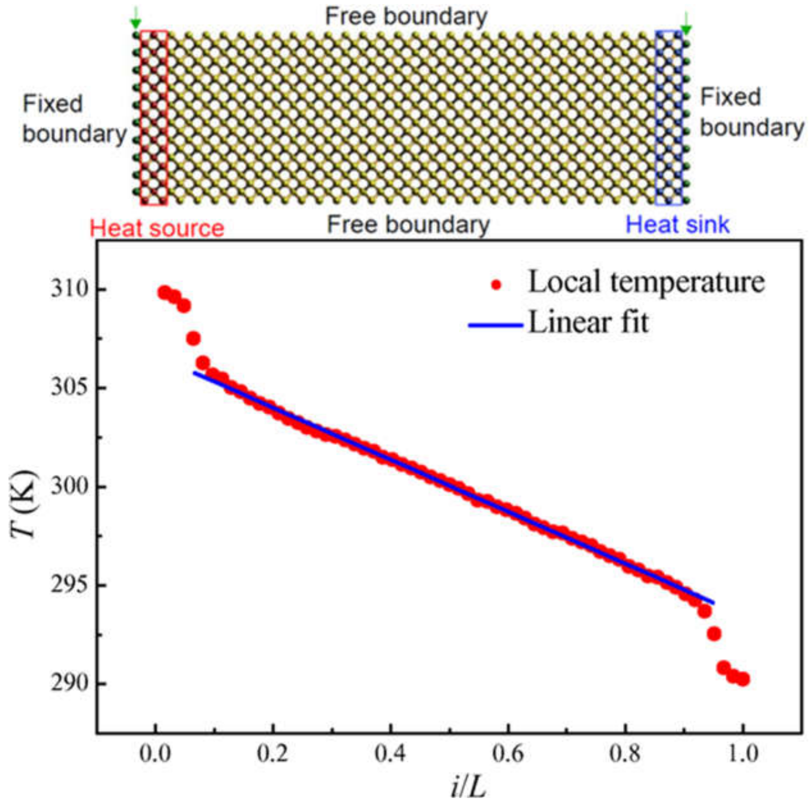

Supplementary Figure 6 | Results from non-equilibrium molecular dynamics simulations. The set-up used in NEMD (top) and steady state temperature profile (bottom).

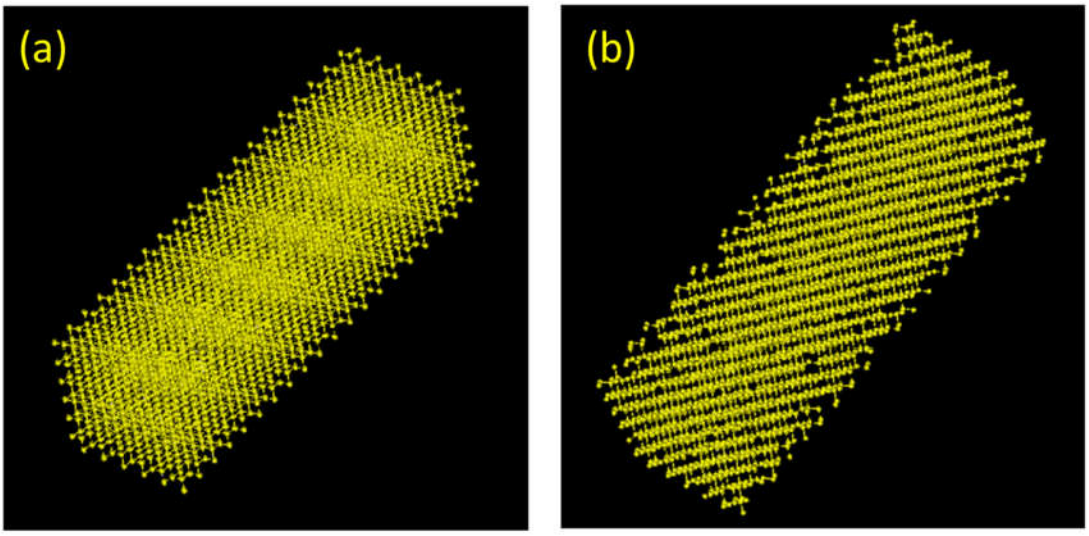

Supplementary Figure 7 | Vacancy effect on thermal conductivity by NEMD. (a) Un-damaged Si nanowire; (b) Si nanowire with vacancies randomly removed.

.

Sample	#1	#2	#3	#4	#5	#6	#7	#8
L_1/nm	300	350	350	400	400	800	400	800
L_2/nm	250	800	650	900	400	500	350	800
Dose (cm^{-2})								
**7.5×10^{16}**	√							
**7×10^{16}**								√
**6.5×10^{16}**								√
**6×10^{16}**								√
**5×10^{16}**		√				√		
**4×10^{16}**						√		
**3.5×10^{16}**							√	
**3×10^{16}**							√	
**2.5×10^{16}**	√	√					√	
**2×10^{16}**							√	
**1.5×10^{16}**				√		√		
**1×10^{16}**		√		√	√			
**5×10^{15}**	√	√	√					
**2.5×10^{15}**		√	√		√			
**1×10^{15}**	√	√	√		√			
**5×10^{14}**	√		√		√			
**2.5×10^{14}**			√					

Supplementary Table 1 | Irradiation conditions for helium ion damaged Si nanowires. All the irradiations were carried out under helium ion energy of 30-36.2keV and current of 0.2-0.7pA. The irradiated length (L_1) and distance (L_2) were different for each nanowire, as well as the irradiated dose.

Supplementary Note 1 | Damage created by helium ions

In the nuclear stopping process, primary energetic helium ions can knock off Si host atoms from their lattice positions. If the secondary knocked-off Si gains enough energy, it can in turn displace other host Si atoms from their lattice positions. This cascade process continues until the residual energy is smaller than the displacement energy. During this process, a large number of displaced atoms (or Frenkel pairs, each of which is composed of a vacancy and a nearby interstitial) are created along the track of the incoming ion[2]. The stopping range and displaced atoms can be calculated by Monte Carlo simulations based on a binary collision approach (TRIM/SRIM[3]). To mimic our experiment, a 36.2 keV helium ion beam and a 160 nm thick Si film is used in the simulation, and the results are shown in Supplementary Fig. 2. The simulation shows that the damage created by helium ions (the red and green dots in Supplementary Fig. 2 (a)) is discrete rather than continuous. This is because of the light helium ions, which create point and point-like defects rather than large damage clusters, the latter typically resulting from heavy implanting ions[4]. Moreover, the defect profile is relatively spatially uniform (Supplementary Fig. 2 (b)), unlike the case for thick Si substrates, in which a well defined peak exists at the end of range. This is because of the small thickness of Si film and high ion beam energy. Lastly, most of the helium ions penetrate the thin film, leaving only 2% of the helium ions remaining inside the sample. Since each helium ion creates 33 vacancies on average, the number of residual helium atoms is much smaller than the number of damaged lattice sites.

From Supplementary Fig. 2 (b), the silicon vacancies induced by each helium ion are linear with its travelling routes and this relation can be approximated as

$$v(\theta) = 0.00078L(\theta) + 0.147 \quad (1)$$

where $v(\theta)$ is the vacancy created per ion at unit length, $L(\theta)$ is each helium ion actual travelling length and θ is the angle as shown in Fig. 2 (C).

The total vacancies for each ion are the integral of Equation 1, which is

$$V(\theta) = 0.00039\mathrm{L}^2(\theta) + 0.147\mathrm{L}(\theta) + \mathrm{C} \quad (2)$$

where C is a constant. Considering the case when $L(\theta)$ is 0, there would be no vacancies generated, thus this formula is reduces to

$$V(\theta) = 0.00039\mathrm{L}^2(\theta) + 0.147\mathrm{L}(\theta). \quad (3)$$

From Supplementary Fig. 2(C), the actual travelling length of each ion is

$$L(\theta) = 2 \times \frac{\phi}{2} \times \frac{\cos(\theta)+\cos(\theta+d\theta)}{2} = \phi cos(\theta) \quad (4)$$

And the element for integration in the horizontal direction, $dW_{solid}(\theta)$is then,

$$dW(\theta) = \frac{\phi}{2} \times \sin(\theta + d\theta) - \frac{\phi}{2} \times \sin(\theta) = \frac{\phi}{2} cos(\theta) d(\theta) \quad (5)$$

Thus, assuming that 10% of the Si vacancies created by helium ions can survive, the number of vacancies remaining is a function of dose. Combining the equations 1-5, we obtain

$$\frac{2 \times \int_0^{\frac{\pi}{2}} V(\theta) \cdot dW(\theta) \times length}{\frac{\pi}{4} \times \phi^2 \times length} \times 10\% \times dose \approx \frac{0.20}{10^{-7}(cm)} \times 10\% \times dose(\mathrm{cm}^{-2})$$

$$= 2.0 \times 10^5(\mathrm{cm}^{-1}) \times dose(\mathrm{cm}^{-2}) \quad (6)$$

Supplementary Note 2 | Calculation of the phonon - point defect scattering rate

According to kinetic theory of phonon gas, thermal conductivity (κ) can be calculated as

$$\kappa = \frac{1}{N}\sum_{q,s} C(q,p)v^2(q,p)\tau(q,p) \quad (7)$$

where *C*, *v*, and τ are volumetric specific heat, group velocity, and phonon lifetime for the phonon mode with wavevector *q* and polarization *p*; *N* is the number of q-points.

The group velocity component perpendicular to the axis is calculated using the following formula for the phonon mode (q, p),

$$v_{\perp \mathrm{q,p}} = \sqrt{v_{\mathrm{x,q,p}}^2 + v_{\mathrm{y,q,p}}^2} \quad (8)$$

where $v_{\mathrm{x,q,p}}$ and $v_{\mathrm{y,q,p}}$ are the *x* and *y* component of group velocity of phonon mode (*q*, *p*), which is computed using the following formula,

$$v_{\mathrm{q,p}} = \frac{\mathrm{d}\omega_{\mathrm{q,p}}}{\mathrm{dk}} \quad (9)$$

The phonon lifetime is estimated from different phonon scattering mechanisms by using Matthiessen's rule, namely,

$$\tau^{-1} = \tau_{\mathrm{a}}^{-1} + \tau_{\mathrm{i}}^{-1} + \tau_{\mathrm{b}}^{-1} + \tau_{\mathrm{d}}^{-1} \quad (10)$$

where τ_{a}^{-1}, τ_{i}^{-1}, τ_{b}^{-1}, and τ_{d}^{-1} are phonon scattering rates (inverse of phonon lifetime) due to anharmonic phonon-phonon, phonon-isotope, phonon-boundary, and phonon-defect scattering, respectively. τ_{a}^{-1} is widely assumed to be proportional to ω^2. The isotope scattering rate is of the form, $\tau_{\mathrm{i}}^{-1} = C\omega^4$, in which *C* is analytically calculated to be $1.32\mathrm{x}10^{-45}$ s^3 for silicon[5,6]. The boundary scattering rate, τ_{b}^{-1}, is estimated by

$$\tau_{\mathrm{b}}^{-1} = \frac{1-\mathrm{m}}{1+\mathrm{m}}\frac{3\pi \mathrm{v}_{\perp}}{4\mathrm{d}}, \quad (11)$$

where $v_{\perp}$ is the group velocity perpendicular to the transport direction[7], and d is the diameter of the Si nanowire (d=160 nm); while m represents the smoothness of surface of silicon nanowire, which is equal to 1 for silicon nanowire with a perfectly smooth surface, and 0 for a completely roughened surface with a fully diffusive boundary. As for the phonon-defect scattering rate, it follows the relationship $\tau_{\mathrm{d}}^{-1} = D\omega^4$. Therefore, the total phonon lifetime can be modelled as

$$\tau^{-1}(\omega) = A\,\omega^2 + C\,\omega^4 + \frac{3\pi v_{\perp}}{4d} + D\,\omega^4 \qquad (12)$$

where A and D are fitting parameters. In our fitting procedures, the phonon dispersion is computed from first principle density functional theory as implemented in Quantum ESPRESSO[8]. The Perdew, Burke and Ernzerhof functional (PBE)[9] and norm-conserving pseudopotential are adopted to describe the exchange-correlation interaction between electrons and the interaction between electrons and ions, respectively. The primitive cell of Si is fully relaxed by a k-point mesh of 25×25×25. The energy cutoff is chosen to be as high as 50 Ry for the expansion of wavefunction by using plane-wave basis sets. After structure relaxation, the force constants are calculated by using finite displacement method with a supercell of 4×4×4. The phonon dispersion is further obtained by diagonalizing dynamic matrix.

We firstly fit the parameter A from the measured thermal conductivity of undamaged Si nanowire. The best fitting gives rise to 5.39×10^{-17} s for A, which is close to widely used value for bulk silicon, i.e., 3.28×10^{-17} s[6]. Afterwards, the parameter D is fitted for each helium ion dose. We do two different fits—one with and one without the boundary scattering term. The results are shown in Figure 3(b) in the main text.

Supplementary Note 3 | Non-equilibrium molecular dynamics (NEMD) simulations for effect of point defects

In the simulation, Si nanowires with various cross-sections of 3×3, 6×6 and 9×9 unit cells (0.543 nm per unit cell) and fixed length of 20 unit cells were used. Stillinger–Weber (SW) potential[10] was used to derive the force term. Velocity Verlet algorithm is used to integrate Newton's equations of motion, with a time step of 0.5 fs. Fixed boundary condition is used at the two ends of nanowires in the longitudinal direction, and free boundary condition is applied to the atoms on the nanowire surface in the transverse direction (Supplementary Fig. 6). Next to the fixed boundary layers, Langevin heat reservoirs[11] with different temperatures are applied as heat source and sink. Thermal conductivity is computed according to Fourier's law: $\kappa = -J / \nabla T$, where J is the heat flux and ∇T the temperature gradient. The heat flux is calculated as the energy induced by the heat bath per unit time per unit area $J = \frac{1}{S} \sum_{i \in \text{heat bath}} \boldsymbol{F}_i \cdot \boldsymbol{v}_i$, where S is the cross sectional area, and $\boldsymbol{F}_i$ and $\boldsymbol{v}_i$ are the force and the velocity of the ith atom in the heat bath. The local temperature is defined as $T = \frac{1}{3Nk_B} < \sum_{i=1}^{N} m_i \boldsymbol{v}_i \cdot \boldsymbol{v}_i >$, where N is the number of atoms in the local bin, k_B is the Boltzmann constant, and the angular bracket denotes the ensemble average. NEMD simulation is performed long enough to ensure steady state is reached where the heat flux and temperature profile are time independent. The temperature gradient is calculated based on the linear regression analysis of the temperature profile in the middle (Supplementary Fig. 6). The Si nanowire with vacancies is modeled by randomly removing some Si atoms (up to 20%) and then removing the single-bonded atom pairs (Supplementary Fig. 7). For a given vacancy concentration, the final results are averaged over eight different realizations.

Supplementary References

1 Hochbaum, A. I. *et al.* Enhanced thermoelectric performance of rough silicon nanowires. *Nature* **451**, 163-167 (2008).

2 Caturla, M.-J., de La Rubia, T. D., Marques, L. & Gilmer, G. Ion-beam processing of silicon at keV energies: A molecular-dynamics study. *Physical Review B* **54**, 16683 (1996).

3 Ziegler, J. F., Ziegler, M. D. & Biersack, J. P. SRIM – The stopping and range of ions in matter (2010). *Nuclear Instruments and Methods in Physics Research Section B: Beam Interactions with Materials and Atoms* **268**, 1818-1823 (2010).

4 Bernas, H. *Materials Science with Ion Beams*. Vol. 116 (Springer, 2010).

5 Asen-Palmer, M. *et al.* Thermal conductivity of germanium crystals with different isotopic compositions. *Physical Review B* **56**, 9431-9447 (1997).

6 Mingo, N. Calculation of Si nanowire thermal conductivity using complete phonon dispersion relations. *Physical Review B* **68**, 113308 (2003).

7 Li, W. & Mingo, N. Alloy enhanced anisotropy in the thermal conductivity of Si_xGe_{1-x} nanowires. *Journal of Applied Physics* **114**, 054307, doi:10.1063/1.4817523 (2013).

8 Paolo, G. *et al.* QUANTUM ESPRESSO: a modular and open-source software project for quantum simulations of materials. *Journal of Physics: Condensed Matter* **21**, 395502 (2009).

9 Perdew, J. P., Burke, K. & Ernzerhof, M. Generalized Gradient Approximation Made Simple. *Physical Review Letters* **77**, 3865-3868 (1996).

10 Stillinger, F. H. & Weber, T. A. Computer simulation of local order in condensed phases of silicon. *Physical Review B* **31**, 5262 (1985).

11 Chen, J., Zhang, G. & Li, B. W. Molecular Dynamics Simulations of Heat Conduction in Nanostructures: Effect of Heat Bath. *Journal of the Physical Society of Japan* **79**, 074604 (2010).
